# A Cross-sectional Study of the Myths Regarding Room Ventilation in Puerperium Among Saudi Women

**DOI:** 10.7759/cureus.5925

**Published:** 2019-10-16

**Authors:** Zaheera Saadia

**Affiliations:** 1 Obstetrics and Gynecology, Qassim University College of Medicine, Buraidah, SAU

**Keywords:** room ventilation, postpartum practices, puerperium, traditions

## Abstract

Background

Despite the advances in medical health care services, people still believe and follow their traditions. Some of the traditional postpartum practices are harmful to women's health contrary to the common belief. The objective of this study is to investigate the prevalence of myths regarding room ventilation (MRV) among Saudi women.

Methods

We conducted an observational cross-sectional study on 355 women in the postpartum period from the outpatient clinic of Mother and Child Hospital (MCH), Al-Qassim, Saudi Arabia. The investigators interviewed all the study participants to fill the proformas and collect all the study data. We analyzed the study data to estimate the prevalence of MRV in our sample as well as the factors associated with MRV.

Results

Most of our study participants were above 30 years (45%), multigravida (85%), and went through a cesarean section (78%). There was a significant association between MRV and education (P<0.001), occupation (P<0.001), and parity (P<0.001) but not with age (P=0.136). The prevalence of MRV dropped from 80% in women who had primary education only to 13% in women who had high school and college education. Also, it differed according to the participant's occupational status. MRV was prevalent in 56% of housewives vs. only 17% of the employed women. Moreover, it showed a substantial change with parity status. MRV increased from 23% in primiparous to 53% in multigravida.

Conclusion

Our results showed that the MRV is highly prevalent in the Saudi Arabia community and higher levels of education were negatively associated with the beliefs in the MRV. These findings highlight the importance of proper health education of pregnant women as well as the need for a prompt response from the Ministry of Health towards the MRV to eliminate it and spread the proper postpartum health care practices.

## Introduction

Puerperium or postpartum period is the period that follows the delivery of the placenta and lasts around six weeks [1]. Physiological changes occur in the female body during this period that aims to restore its normal shape and physiological functions of the non-pregnant state [1]. These physiological changes include involution of the uterus and cervix, endometrium regeneration, marked diuresis in the first 48 hours with other urinary tract changes, reduction of the body weight, breast preparation for lactation, and regaining the fertile state [2-3].

Women in the postpartum period need to follow the proper health care practices to regain their strength and avoid postpartum complications as infection, sepsis, thromboembolism, excessive bleeding, constipation, and breast problems [4]. Despite the progress and advances in medical health care services and multiple awareness campaigns to the mothers during pregnancy and lactation, people still have their postpartum practices and traditions that vary according to their country and religion [5]. The postpartum practices vary according to country, religion, economic state, and education level. There is a wide range of postpartum practices among the different populations across the world. Some of these practices are the month among Chinese population [6-7], encouraging hot food among Indians [8], purification bath "ghusl" after stoppage of bleeding among Muslim women [9], prevention of mechanical breastfeed pump usage among orthodox Jewish women [10], initiation of breastfeeding after two days among Hindu families in India [11], and bounding of the baby's and mother's abdomen to hasten the stomach and prevent constipation among Muslim families [12].

Some of the prevailing postpartum practices are just myths and either harm the mother's health or have no benefit at all. In our previous article by Saadia et al. [13], we studied the prevailing myths among Saudi women in the postpartum period [13]. One of these myths was the myth of room ventilation (MRV), which was a common postpartum practice among Saudi women. MRV means that women do not ventilate their rooms, keep the doors and windows closed, do not use fans or air conditioners, and remain confined to the closed spaces.

To the best of our knowledge, no previous studies in the literature have described the prevalence of the MRV in the population of Saudi Arabia. Therefore, in this study, we aim to investigate the prevalence and demographic determinants of postpartum MRV among Saudi women. This study is considered the first study to investigate such practice, so it will serve as a pilot for any future research in the same field.

## Materials and methods

We followed the Strengthening the Reporting of Observational Studies in Epidemiology (STROBE statement) guidelines when reporting this observational study (available here: https://strobe-statement.org).

Study design

We conducted an observational cross-sectional study. The details of the study design and methodology were previously published by Saadia et al. [13]. This study was approved by the ethics committee of Qassim University, the Kingdom of Saudi Arabia (KSA) (approval number 201711433). All participants gave informed consent prior to participation in the study.

Study participants

We recruited females in the postpartum period from the outpatient clinic of Mother and Child Hospital (MCH), Al-Qassim, Saudi Arabia during their routine six weeks follow-up from January to December 2011. Verbal consent was given to all study participants before the beginning of the study. All of our study participants were from the Al-Qassim region.

Data collection

Our proformas were filled out by the study investigators. The proforma was tested in a pilot study by a sample size of 20 participants. We collected the following data from each participant: age, education level, occupation, parity, mode of delivery, and room ventilation practice. Then, the collected data were classified into categories. To avoid bias, we did not include the participant name or identification number to keep the study data unidentified. Data of the pilot evaluation were not included in the final analysis of this study.

Data analysis

We used Statistical Package for the Social Sciences; version 23 (SPSS Inc., Chicago, IL) for the analysis of the study data. We used Chi-square (χ²) test to investigate the relationship between room ventilation practice and the demographic variables. Also, contingency tables were used to show the frequency and correlation of room ventilation with demographic variables. A P value below 0.05 was considered for statistical significance.

## Results

Participants characteristics

We interviewed a total number of 360 women for our study, and only 355 women were included in our analysis after excluding five women because of incomplete data. Most of our study participants were above 30 years old (45.4%) and attended the primary or Middle school (67.6%). Only 43 (12.1%) of them had college or above education. Most of them were housewives 289 (81.4%), and 47 (13.2%) were working in the teaching field. Most of the participants were multiparous; gravida (G) 2-4 were 155 (43.7%), and G5 or above were 148 (41.7%), only 52 of them (14.6%) were primiparous. Women who had normal vaginal delivery were only 51 (14.4%), and the rest of them went through cesarean section 276 (77.7) or instrumental delivery 28 (7.9%). The summary of the participants' demographics is shown in Table 1.

**Table 1:** The characteristics of the study participants Table 1 shows the characteristics of the study participants [13].

Variables	Categories	Frequency	%
		N=355	
Age	Below 25	74	20.8
	25-30	120	33.8
	Above 30	161	45.4
Education	Primary or below	127	35.8
	Middle school	113	31.8
	High school	72	20.3
	College or above	43	12.1
Occupation	Housewives	289	81.4
	Teaching	47	13.2
	Professional/trade/business	8	2.3
	Government Service	11	3.1
Parity	Primiparous	52	14.6
	G2-4	155	43.7
	G5 or above	148	41.7
Mode of delivery	Cesarean section	276	77.7
	Instrumental delivery	28	7.9
	Normal delivery	51	14.4

The association between MRV and population characteristics

The Chi-square test for independence showed a significance association between MRV and education (χ23 = 115.44, P<0.001), occupation (χ21 = 33.37, P<0.001), and parity (χ22 = 20.96, P<0.001). On the other hand, it showed a non-significant result for Age (χ22 = 4; P=0.136) (Table 2).

**Table 2:** Chi-square test for independence summary for all variable combinations Note: The teacher, professional, and government worker categories were combined due to the small sample size. MRV: myth of room ventilation.

	Demographic Variable	Chi-Square	df	p
MRV practice (no ventilation)	Age	4.00	2	0.136
	Education	115.44	3	0.000
	Occupation	33.37	1	0.000
	Parity	20.96	2	0.000

Most of the participants who were below 25 years did not ventilate the room during puerperium (58%). On the contrary, most women above 30 years ventilated the room (56%) and from 25 to 30 years almost had an equal distribution. Most women who attended primary education only did not ventilate the room in the puerperium (80%). Contrarily, almost all of the women who attended high-school or college ventilated the room during puerperium (96%) and (72%), respectively. Also, most of the housewives did not ventilate the room (56%), while most of the employed women ventilated the room (83%). In the same way, the majority of primiparous women ventilated the room (77%), while G 2-4 did not ventilate the room (60%). The summary of the results is shown in Table 3.

**Table 3:** Contingency table for room ventilation and the demographic variables

Demographic Variables		n	Do not ventilate the room	Ventilate the room
Age	Below 25	74	58.10%	41.90%
	25-30	120	49.20%	50.80%
	Above 30	161	44.10%	55.90%
Education	Primary or below	127	80.30%	19.70%
	Middle school	113	49.60%	50.40%
	High school	72	4.20%	95.80%
	College and above	43	27.90%	72.10%
Occupation	Housewife	289	56.10%	43.90%
	Employed	66	16.70%	83.30%
Parity	Primiparous	52	23.10%	76.90%
	G2-4	155	59.40%	40.60%
	G5 and above	148	46.60%	53.40%

## Discussion

In our previous study, we discussed the dietary practices during puerperium among Saudi women [13]. In the present study, we explored the prevalence of MRV tradition in puerperium in Saudi culture and the factors affecting the myth of RV during puerperium. Our study reveals the wrong practices and myths regarding room ventilation which might help in the development of a productive health awareness program in Saudi Arabia. 

Our study showed that MRV was significantly associated with education (P<0.001), occupation (P<0.001), and parity (P<0.001) but not with age (P=0.136). The impact of education on the MRV was evident in this study; 80% of women who attended primary education only believed in the MRV and therefore, they did not ventilate the room in the puerperium. On the contrary, 96% and 72% of women who attended high-school or college ventilated the room during the puerperium, therefore, women with higher levels of education did not practice the MRV. Employment was another factor since most of the employed women ventilated the room compared to housewives (83% vs. 56%).

There are previous studies that discussed other postpartum practices in different countries across the world. Doing the month or "sitting month" or "Zuo Yue Zi" is a postpartum tradition that is very common in China. During that month, the women are advised to stay at home with no outdoor activities, eat hot food staff and avoid cold food like fruits and vegetables, and avoid bathing and hair washing [14-16]. Other studies discussed the postpartum practices in Latin American countries as la cuarentena tradition. La cuarentena is a six-week period, during which the mother restrains herself from sex and dedicates her time for breastfeeding and taking care of the new baby [17-18]. Homebirth in the Netherlands is also one of the traditions that had been discussed in the literature [19]. Dutch women have the highest percentage among the new world countries in home birth “The Netherlands has the highest percentage of home births in the Western world,” said Sjaak Toet, the chairman of the Dutch Association of Midwives (KNOV) [20]. Burying the placenta after delivery is one of the most exotic postpartum traditions in the world. It occurs in some parts of Australia and Bali island, Indonesia [21-22].

Postpartum MRV in Middle-East countries had never been discussed before, that is why we are aiming to show its prevalence in our community in Saudi Arabia. Being the first to study MRV in the region is the main strength point of this study; our study will be considered as a pilot for other researchers to investigate this practice and fight this traditional postpartum malpractice in the region.

In the parity category, the prevalence of MRV practice was 23% in primiparous, almost 60% in G 2-4 women, and 46% among women above G5. The results of the parity category were a little unexpected as we may say. It increases from 23% in primiparous to 47% in G5 and above. This increase in the multigravida group can be explained by the multiparous mothers feel more relief about their pregnancy and depend on their mothers' traditions and practices for their health care. In reverse, the primiparous women are more cautious and seek the best medical health care for themselves and for her first baby that is why a small percentage of them follow their community practices. We cannot refer our results to the economic status of the participants, either housewife or employed, as the gross domestic product per capita is fairly good and access to medical health care is free for all the population [23].

The postpartum practices were created based on some fictional beliefs. As an example, la cuarentena practice was made to increase the bond between the mother and her baby and to protect the mother from any contagious infection [5]. When asked about the reasons for MRV practice, people justified the MRV by their beliefs that confining to one place, closing the doors, and not using air conditioning will protect the mother from any contagious infections and will protect the mother from body aches and pains. Contrary to this belief, MRV practice can cause harm to women's health; it can increase (1) the incidence of infection especially respiratory tract infections due to the unconditioned air, (2) psychological troubles due to room confinement, (3) deep venous thrombosis due to prolonged recumbency, and (4) gastrointestinal tract problems as constipation.

Our study results are limited by some factors: (1) Small sample size (only 355 participants). (2) Being a single-center study such as ours, which is hospital-based, and all of our participants being selected from MCH (Al-Qassim, Saudi Arabia), we cannot generalize our results for the whole community. Despite this, we believe that our sample results are very close to the true prevalence in the population. (3) Study design - as our study is a cross-sectional study, we cannot detect the true reasons behind this postpartum malpractice and traditions. (4) We could not compare the prevalence of MRV across the different areas of Saudi Arabia (i.e., rural versus urban regions) since our study was confined to a single center.

We recommend conducting cohort studies with a follow-up period long enough to study the effect and outcomes of postpartum RV practice on mother and child health. Also, we recommend conducting multi-center cross-sectional studies with a large sample size of >1000. Our study and the other studies that follow our lead will help to identify the needs to create a governmental educational program and awareness campaigns to improve the knowledge and the traditional myths of the common people (especially women below high school level) about pregnancy and the postpartum period. Moreover, we highly recommend conducting a large survey to study all the traditional postpartum practices in the Middle-East region.

## Conclusions

Our results showed that MRV is highly prevalent in the Saudi Arabia community and higher levels of education were negatively associated with the beliefs in MRV. These findings highlight the importance of proper health education among pregnant women as well as the need for a prompt response from the Ministry of Health towards MRV to eliminate it and spread the proper postpartum health care practices.

## References

[1] Pessel C, Tsai MC (2012). The normal puerperium. Current Diagnosis & Treatment: Obstetrics & Gynecology, Eleventh Edition.

[2] Harrison JM (2000). Physiological changes of the puerperium. Br J Midwifery.

[3] Willms AB, Brown ED, Kettritz UI, Kuller JA, Semelka RC (1995). Anatomic changes in the pelvis after uncomplicated vaginal delivery: evaluation with serial MR imaging. Radiology.

[4] (2019). Postpartum problems: sex, skin, urinary, and other post-pregnancy issues. https://www.webmd.com/parenting/baby/features/postpartum-problems.

[5] Dennis CL, Fung K, Grigoriadis S, Robinson GE, Romans S, Ross L (2007). Traditional postpartum practices and rituals: a qualitative systematic review. J Womens Health.

[6] Pillsbury BLK (1978). “Doing the month”: confinement and convalescence of Chinese women after childbirth. Social Science & Medicine Part B: Medical Anthropology.

[7] Tung WC (2010). Doing the month and Asian cultures: implications for health care. Home Health Care Manag Pract.

[8] Choudhry UK (1997). Traditional practices of women from India: pregnancy, childbirth, and newborn care. J Obstet Gynecol Neonatal Nurs.

[9] Fonte J, Horton-Deutsch S (2005). Treating postpartum depression in immigrant Muslim women. J Am Psychiatr Nurses Assoc.

[10] Chertok I (1999). Relief of breast engorgement for the Sabbath-observant Jewish woman. J Obstet Gynecol Neonatal Nurs.

[11] Gatrad AR, Ray M, Sheikh A (2004). Hindu birth customs. Arch Dis Child.

[12] Nahas VL, Hillege S, Amasheh N (1999). Postpartum depression: the lived experiences of Middle Eastern migrant women in Australia. JMWH.

[13] Saadia Z, Roshdy S, Sagir F, Abidin S (2013). Dietary practices of Saudi women during puerperium. J Obstet Gynaecol Res.

[14] Liu N, Mao L, Sun X, Liu L, Chen B, Ding Q (2006). Postpartum practices of puerperal women and their influencing factors in three regions of Hubei, China. BMC Public Health.

[15] Wang XL, Wang Y, Zhou SZ, Wang J, Wang JL (2007). Puerperal practice pattern in a rural area of north China [Article in Chinese]. Beijing Da Xue Xue Bao.

[16] Chang SH, Hall WA, Campbell S, Lee L (2018). Experiences of Chinese immigrant women following "Zuo Yue Zi" in British Columbia. J Clin Nurs.

[17] Acosta DF, de Oliveira Gomes VL, da Costa Kerber N, da Costa CFS (2012). Influências, crenças e práticas no autocuidado das puérperas [Article in Spanish]. Escola de Enfermagem da USP.

[18] Waugh LJ (2011). Beliefs associated with Mexican immigrant families' practice of La Cuarentena during postpartum recovery. J Obstet Gynecol Neonatal Nurs.

[19] van der Kooy J, Birnie E, Denktas S, Steegers EAP, Bonsel GJ (2017). Planned home compared with planned hospital births: mode of delivery and perinatal mortality rates, an observational study. BMC Pregnancy Childb.

[20] Berg Svd (2019). Why the Dutch cherish home births. Expatica.

[21] Burns E (2014). More than clinical waste? Placenta rituals among Australian home-birthing women. J Perinat Educ.

[22] (2019). Bali: the sacred placenta. http://givethehug.blogspot.com/2013/09/the-sacred-placenta.html.

[23] (2019). Saudi Arabia GDP per capita, 2019. https://tradingeconomics.com/saudi-arabia/gdp-per-capita.

